# An outbreak of Coxsackievirus A6–associated hand, foot, and mouth disease in a kindergarten in Beijing in 2015

**DOI:** 10.1186/s12887-018-1253-1

**Published:** 2018-08-21

**Authors:** Jie Li, Rong Zhu, Da Huo, Yiwei Du, Yuxiang Yan, Zhichao Liang, Yanxia Luo, Yang Yang, Lei Jia, Lijuan Chen, Quanyi Wang, Yan He

**Affiliations:** 1 Department of Epidemiology and Biostatistics, School of Public Health, Capital Medical University, No. 10 Xitoutiao, You’anmen Wai, Fengtai District, Beijing, 100069 People’s Republic of China; 2 Institute for Infectious Disease and Endemic Disease Control, Beijing Center for Disease Prevention and Control, No.16, Hepingli Middle Street, Beijing, 100013 People’s Republic of China

**Keywords:** Hand foot and mouth disease, Disease outbreak, Coxsackievirus A6, Onychomadesis

## Abstract

**Background:**

Coxsackievirus A6 (CVA6) is one of the major agents to cause hand, foot and mouth disease (HFMD) outbreaks globally. The objective of this study is to investigate the epidemiologic and clinical manifestations of CVA6 outbreak, and thus guide the diagnosis and treatment of the disease, as well as disease prevention.

**Methods:**

An HFMD outbreak in a kindergarten was reported to Shijingshan District Center for Disease Control and Prevention (SCDC) on November 2, 2015 in Beijing, China. Epidemiological investigation was conducted. We performed a nine-week follow-up study to collect and analyze the clinical manifestations of HFMD cases.

**Results:**

The outbreak yield 56 (15.7%) clinical diagnosed HFMD cases out of 357 registered children in the kindergarten with the mean age of 3.5 years old. This outbreak lasted for three days and ceased after initiating infectious disease controlling procedures, including periodical suspension of the kindergarten activities, environmental disinfection, and family health education. Fifty-one cases were followed for nine weeks. The positive rate of clinical manifestations of rash, fever, desquamation, pigmentation and onychomadesis were 100.0%, 84.3%, 68.6%, 17.6% and 43.1%, respectively. Children developed desquamation within the first 4 weeks after disease onset and developed onychomadesis between the 3th and 8th week after disease onset. Children with desquamation had 9.3 (95%CI: 1.836–47.437) times higher odds of developing onychomadesis compared to those without this manifestation. Ten out of 14 collected samples were CVA6 positive, and five positive samples shared a high degree of similarity in the VP1 nucleotide and amino acid sequences (99.9–100.0% and 100%).

**Conclusion:**

This HFMD outbreak was caused by CVA6, featured with delayed symptoms. Emerging CVA6-associated HFMD and its delayed symptoms should be paid more attention to reduce outbreaks and provide more information to doctors and parents.

**Electronic supplementary material:**

The online version of this article (10.1186/s12887-018-1253-1) contains supplementary material, which is available to authorized users.

## Background

Hand, foot and mouth disease (HFMD) is an infectious disease characterized by clinical symptoms of vesicular rashes and stomatitis of the oral mucosa affecting patients’ hands, feet and occasionally the buttocks. The most susceptible patient population of HFMD is children less than 5 years old. HFMD is caused by viruses belonging to the species Enterovirus A or B, genus Enterovirus, family Picornaviridae, among which, human enterovirus 71 (EV-A71) and Coxsackievirus A16 (CVA16) were the most prevalent pathogens in previous publications [1–3]. Only EV-A71 is almost exclusively associated with severe disease.

From 2012 onward, Coxsackievirus A6 (CVA6) has been reported as an emerging pathogen of the HFMD epidemic globally. In 2013, the etiological surveillance system in Beijing and other cities in China identified a sudden rise of CVA6 positive rate, which turned out to be one of the predominant pathogens of HFMD [4–7]. Since then, CVA6 has spread across mainland China and drawn serious public health attention.

In 2015, there were in total six HFMD outbreaks occurred in the kindergartens in Beijing. The number of cases ranged from 11 to 14 among the five of them, and remaining one had 56 cases. This outbreak occurred in a kindergarten in Shijingshan district in Beijing. There were 357 children and 51 school staffs in this kindergarten. The children were divided and assigned to 11 classes (average class size was 32). Daily extra-curricular activities were held, in which children from different classes attended lessons together. We performed a further investigation on this large-scale HFMD outbreak in this kindergarten and found that it was caused by CVA6 infection.

In this study, we aimed to collect and summarize epidemiological and clinical evidence of CVA6 infected HFMD, and thus to support and guide the diagnosis, treatment, prevention and control of the disease.

## Methods

### Case definition and outbreak definition

A clinical case of HFMD was defined as presenting with oral ulcers and maculopapular or vesicular rash distributed over the hands, feet and buttocks, with or without fever. Cases were classified as severe if they presented any cardiopulmonary complications (pulmonary edema, pulmonary hemorrhage, or cardiorespiratory failure) or neurological complications (aseptic meningitis, encephalitis, encephalomyelitis, acute flaccid paralysis, or autonomic nervous system dysregulation) [8].

According to the “Protocol of control practice for the clustered cases and outbreaks of hand foot and mouth disease” issued by the National Health and Family Planning Commission of China in 2012 [9], an HFMD outbreak was defined as ≥10 cases of HFMD occurring in a kindergarten or school within a week. Once an outbreak is reported from a kindergarten or a school, the health practitioners from local CDC will have a risk assessment on the situation, upon which a school suspension decision will be made. In order to confirm the disease causing agent of the outbreak, at least five samples are required to be collected and analyzed.

### Sample and data collection

Once the district CDC got a report of HFMD outbreak, the epidemiological investigation was conducted immediately. Demographic information (ID, birth date, gender), clinical symptoms (fever, rash, sore throat, desquamation, onychomadesis), and epidemiological data (onset date of HFMD, the date of visiting a hospital, onset date of delayed symptoms, clinical diagnostic result and laboratory test result) and throat swab samples were collected by CDC staffs on the same day of the field investigation with prior written informed consent. The teacher in charge of each classroom was responsible for reporting any new case to the doctors of community healthcare centers who would pay the sick children a home visit, collected information with prior written informed consent, and reported to the local CDC afterwards. Parents or guardians of the sick children were followed up twice by telephone to collect information about the delayed symptoms, such as onychomadesis, desquamation and chromatosis, at 4th and 9th weeks after the onset of HFMD.

### Sample processing mode, nucleotide extraction and virus identification

The specimens were vortexed for 1 min and centrifuged at 4000×g for 20 min before nucleotide extraction. Total nucleotide extraction was performed with MagNA Pure LC Total Nucleic Acid Isolation Kit-Large Volume (ROCHE, Co, USA) by a Roche MagNA Pure LC 2.0 nucleic extraction system (ROCHE, Co, USA) according to the manufacturer’s instructions. EV, EV-A71 and CVA16 were identified using real-time RT-PCR Kit (DAAN Gene, Guangzhou, China) [10]. Samples of non-EV-A71 and non-CVA16 were further identified by detecting the VP1 region according to previously described method [11]. Complete nucleotide sequences of VP1 genes were amplified using previously described specific primers [12]. Polymerase Chain Reaction (PCR) products of complete VP1 genes were purified and sequenced using ABI PRISM310 Genetic Analyzer.

### Phylogenetic analysis of CVA6

To further study the homology of sequences from viruses in this outbreak, sequence analysis of VP1 was carried out using Molecular Evolutionary Genetics Analysis Version 6.0 (MEGA6). The phylogenetic tree was constructed by Maximum likelihood method with bootstrapping of 1000 replicates. A total of 5 CVA6 isolates representing this outbreak were aligned and compared with the corresponding region of CVA6 isolates from HFMD casess through 2009 to 2015 in Beijing.

### Statistical analysis

Statistical analysis was conducted using SPSS17.0 software (IBM SPSS Inc., Chicago, IL, USA). Continuous variables in asymmetric distribution were described using the median with 25th and 75th percentiles. Frequencies and proportions were used for categorical variables. Comparisons of proportions were performed using the Chi-square test. A two-tailed *p* value of less than 0.05 (2-sided significance testing) was considered to be statistically significant.

### Ethics statement

This study was in compliance with the Helsinki Declaration and was approved by the Human Research Ethics Committee of Beijing CDC. The approval number of the local ethics committee was 201,809. Sample collection in this study was agreed by the patient’s guardian with prior written informed consent.

## Results

In 2015, a total of six outbreaks of HFMD occurred in Beijing kindergartens (Table 1). Four out of six outbreaks were caused by CVA6. The remaining two outbreaks were caused by EV71 and CVA16. Among the six outbreaks, the largest one was caused by CVA6, in which 56 cases were identified.

**Table 1:** Epidemiology characteristics of six HFMD outbreaks in Beijing in 2015

ID	District	Classification	Number of Cases	Attack rate (%)	^a^Age (year)	Male/Female	Date	Agent
1	Fangshan	Kindergarten	14	17.7	3.0(2.0, 3.0)	2.5:1	Apr	CVA16
2	Mentougou	Kindergarten	16	6.5	4.0(4.0, 5.0)	1.3:1	Jun	CVA6
3	Fangshan	Kindergarten	22	12.0	3.0(3.0, 3.0)	1.8:1	Jul	EV-A71
4	Fengtai	Kindergarten	11	2.1	5.0(5.0, 5.0)	0.8:1	Jul	CVA6
5	Chaoyang	Kindergarten	14	4.0	4.0(4.0, 4.3)	0.8:1	Sep	CVA6
6	Shijingshan	Kindergarten	56	15.7	3.4(3.7, 4.3)	0.9:1	Oct	CVA6

^a^Presented as the median (p25, p75)

### Epidemiological investigation

This outbreak occurred in a kindergarten in Shijingshan district and was reported on November 2, 2015. In response, Shijingshan district CDC (SCDC) initiated an epidemiological investigation immediately.

**Fig. 1:**
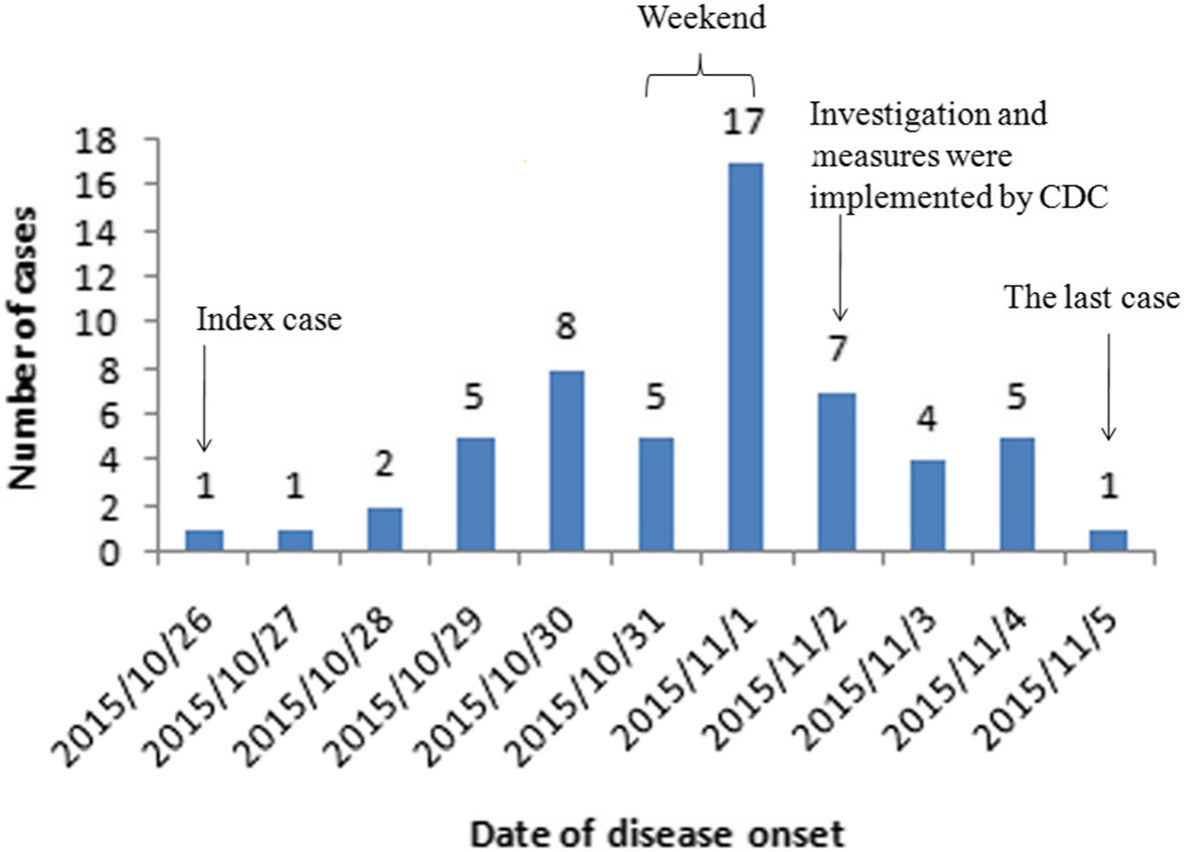
Number of children with HFMD in the outbreak by date of disease onset (*n* = 56)

From October 26th to November 5th, a total of 56 clinically diagnosed cases from 10 classes were reported. The attack rate was 15.7% (56/357). From October 27th to 30th, 16 children from different classes were reported with fever or rash on body. During the following weekend (October 31 and November 1), this outbreak attained the peak and a total of 22 cases were obtained. From November 2nd to 5th, another 17 cases were reported (Fig. 1).

There were 357 children and a health-care doctor in this kindergarten. Every morning, school doctor did regular body checks for each child to see if they present any fever, skin rashes or herpangina. Once a symptom is found, the parents or guardians would be notified to take their children for further medical examination. During the noon break, the same regular body checks would be performed again. The first case in this outbreak appeared on October 26, 2015 a 5-year-old girl who had low-grade fever with no rash on her hands or feet. She felt ill in the afternoon and was taken home by her mother and rested at home for following days. On October 29, her mother found generalized vesicular rashes distributed over her hands and feet and took her to a hospital, where she was diagnosed with HFMD. Her parents did not recall or report exposure to any HFMD cases before diagnosis.

### Outbreak control measures

Infection-control measures, including case isolation, contact medical observation, environmental disinfection, suspension of the kindergarten, and health education, were immediately implemented. All the children diagnosed with HFMD were isolated at home for at least 10 days until recovered. Kids could return to kindergarten only if the community health care center proved that they have completely recovered. Classrooms, indoor playground, bathrooms, kitchens and recreation facilities were cleaned and disinfected thoroughly. Based on the fact that children from 10 out of the 11 classes were infected (according to the clinical diagnosis) and HFMD was prone to spread rapidly, the kindergarten was advised to take a periodical suspension for 10 days. The health practitioners from local CDC and health care centers did health education for children and their parents, as well as the kindergarten staffs.

### Epidemiological characteristics, clinical manifestations and laboratory test result

During this outbreak, to obtain more detailed information about the prognosis and the delayed symptoms such as desquamation and onychomadesis, we contacted all the HFMD patients in this the outbreak. Five parents of the patients refused, thus, 51(91.1%) volunteered to enroll in this study and were successfully followed up at 4th and 9th week after illness onset. Among the 51 patients, 26 (51.0%) were girls, and the median age was 44 months (IQR 41.0–51.0). Six out of 51 cases (11.8%) had a history of HFMD disease.

All the cases sought for medical treatment and were clinically diagnosed as mild HFMD in seven hospitals and fully recovered without any complications. Epidemiology and clinical presentations of HFMD cases in this study were shown in Table 2. The most common clinical manifestation was skin rash (51/51, 100.0%), followed by fever (43/51, 84.3%). A total of 35 children (68.6%, 35/51) developed desquamation within the 1st and the 4rd week after disease onset. Of the 51 patients, 22 (43.1%) experienced onychomadesis between the 3rd and 8th week after disease onset (Additional file 1). The most common location of onychomadesis on the hands and feet were the thumbs (17/51, 33.3%) and halluces (8/51, 15.7%), respectively. In total, 68 finger nails from 22 kids and 17 ft nails from 8 children were found with onychomadesis. Twelve (54.5%, 12/22) cases showed the symptom of onychomadesis on symmetrical location. Among the 51 patients, two (3.9%) lost all their fingernails.

**Table 2:** Epidemiology and clinical presentations of HFMD cases in this study

Characteristics	N (%)
Sex	
Male	25(49.0)
Female	26(51.0)
^a^Age(months)	44(41.0-51.0)
Fever	43(84.3)
^a^Duration(day)	1.5(1.0-2.0)
^a^The highest temperature(°C)	38.7(38.4–39.2)
≥ 39 °C	12(23.5)
Rash	
Hand	48(94.1)
Foot	37(72.5)
Oral cavity	32(62.7)
Perioral area	19(37.3)
Buttock	16(31.4)
Extremity	6(11.8)
Trunk	5(9.8)
Bullous eruption	2(3.9)
Pigmentation	9(17.6)
Desquamation	35(68.6)
Onychomadesis	22(43.1)
Hands	21(41.2)
Thumb	17(33.3)
Forefinger	10(19.6)
Middle finger	8(15.8)
Ring fingers	5(9.8)
Little finger	3(5.9)
Feet	8(15.7)
The big toe	8(15.7)
The second toe	1(2.0)
The third toe	1(2.0)
^a^Median duration from onset to diagnosis(day)	1(0.0–3.0)
Laboratory test	
^a^WBC (10^9^/L)	9.2(6.8–10.8)
^a^CRP (mg/L)	9.4(8.0–13.5)
^a^Lymphocyte count(10^9^/L)	3.1(2.3–4.0)
^a^Lymphocyte percentage (%)	36.0(24.6–52.4)

WBC, white blood cell; CRP, C-reactive protein

^a^Presented as the median (p25, p75)

Laboratory results were available among 26 cases which were collected by staffs from the health care community center during home visit. The median WBC count, median CRP value, median lymphocyte count and median lymphocyte percentage of cases were 9.2*10^9^/L (6.8–10.8), 9.4 mg/L (8.0–13.5), 3.1*10^9^/L (2.3–4.0) and 36.0% (24.6–52.4), respectively. The WBC counts of 26.9% (7/26), CRP of 37.5% (6/16) and lymphocyte count of 47.6% (10/21) were above the upper limit of the normal range.

Given that onychomadesis was described as a late complication of HFMD and the role of CVA6 in causing onychomadesis was unclear, we attempted to examine factors associated with the manifestations [13, 14]. As shown in Table 3, age, sex, fever, body temperature, pigmentation and median duration from onset to initial diagnosis were not significantly associated with children’s onychomadesis status (*P* > 0.05). Cases with the symptom of desquamation have 9.3 times higher odds (OR = 9.3, 95% CI, 1.836–47.437) of experiencing onychomadesis comparing with those cases without desquamation.

**Table 3:** Factors associated with onychomadesis

	Onychomadesis		OR(95%CI)	*P* ^*a*^
	Yes (%)	No (%)		
Age(month)(*n* = 51)				
36–47	16(72.7)	15(51.7)	0.402(0.123–1.318)	0.128
48–72	6(27.3)	14(48.3)		
Sex (*n* = 51)				
Female	9(40.9)	17(58.6)	2.046(0.664–6.311)	0.210
Male	13(59.1)	12(41.4)		
Median duration from onset to initial diagnosis(days)				
≤ 2	16(72.7)	16(55.2)	0.462(0.140–1.517)	0.199
> 2	6(27.3)	13(44.8)		
Fever(*n* = 51)				
No	5(22.7)	3(10.3)	0.392(0.083–1.860)	0.228
Yes	17(77.3)	26(89.7)		
Temperature(*n* = 43)				
37.3 ≤ *T* < 39 °C	13(76.5)	14(53.8)	0.359(0.092–1.399)	0.133
T ≥ 39 °C	4(23.5)	12(46.2)		
Desquamation(n = 51)				
Yes	20(90.9)	15(51.7)	**9.3 (1.836–47.437)**	**0.003**
No	2(9.1)	14(48.3)		
Pigmentation(n = 51)				
No	16(72.7)	26(89.7)	3.250(0.711–14.851)	0.150
Yes	6(27.3)	3(10.3)		

^a^*P* < 0.05 from the *x*^2^ test was considered to be significant

The bold values means significant difference was obtained between compared groups

### Pathogen detection and phylogenetic analysis

**Fig. 2:**
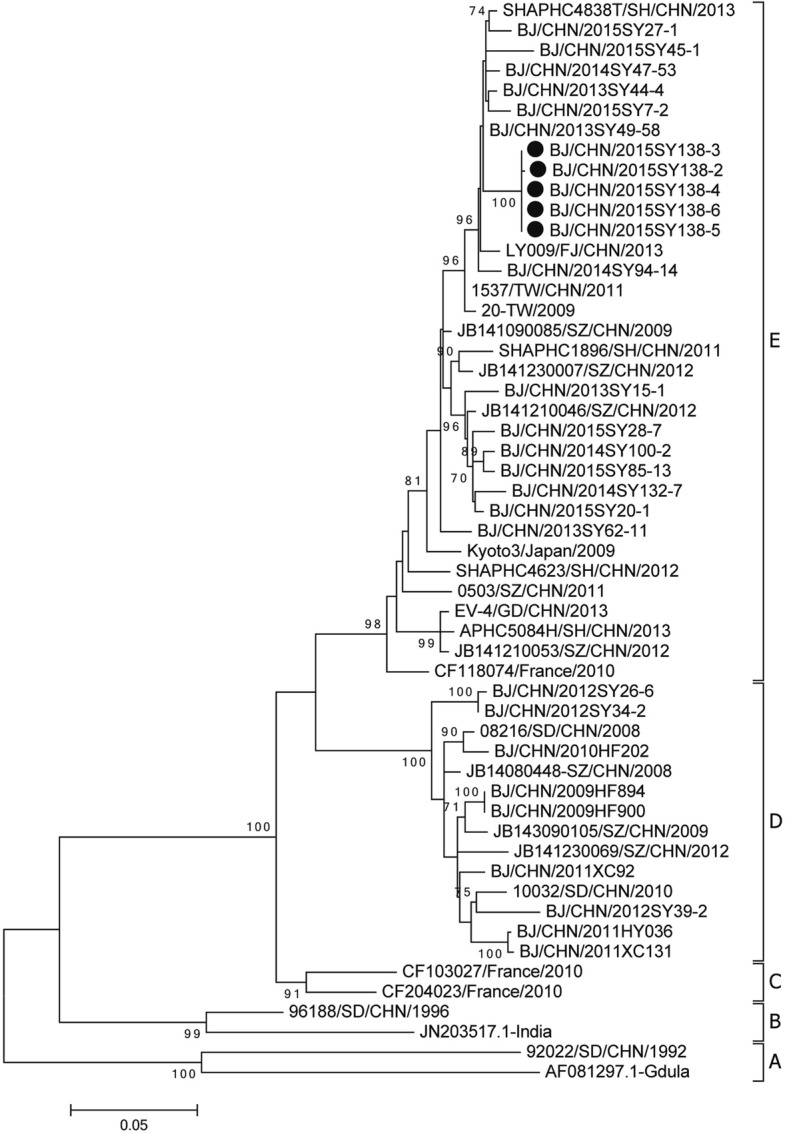
Phylogenetic dendrogram based on the alignment of the complete VP1 gene sequence of CVA6 (915 bp). CVA6 strains isolated from this outbreak were indicated by a solid dot

During the outbreak, parents of 14 cases (including the parents of the index case) agreed to provide samples for pathogen detection. A total of 14 throat samples from patients were analyzed, and 10 (71.4%) samples (including the sample from the index case) were CVA6 positive. Another three samples, including a swab throat from the health-care teacher and two samples from the tableware and the toilet, were CVA6 negative. Among five out of ten CVA6 positive samples, the VP1 sequences were successfully amplified. The similarity in the nucleotide and amino acid sequences among them was 99.9–100.0% and 100%, respectively. We analyzed the VP1 of CVA6 viruses from this outbreak (MG488204, MG488205, MG488206, MG488207, and MG488208) and from previous cases during 2009–2015 in Beijing. All the CVA6 strains were classified into five major groups (A-E) (Fig. 2). CVA6 isolates in Beijing from 2009 to 2012 were classified as group D. CVA6 isolates from 2013 to 2015 were classified as group E. The nucleotide identity for CVA6 strains in this study and from 2009 to 2012, 2013–2014 and 2015 in Beijing was 87.7–89.2%, 97.8–99.3% and 94.2–98.3%, respectively. CVA6 strains (including the CVA6 strain from the index case) from the outbreak in this study were all classified as group E.

## Discussion

According to the etiology surveillance system of Beijing, in 2009, 2011, 2012, and 2014, CVA16 was identified as the main pathogen of HFMD, while in 2010, EV-A71 was the major pathogen. However, in 2013 and 2015, CVA6 surpassed CVA16 and EV-A71 to become the predominant pathogen of HFMD [15]. In our study, CVA6 virus was identified as the dominant causative agent, which is consistent with the epidemic background of HFMD in Beijing during 2015.

Considering the fact that the average incubation period of HFMD ranges from 3 to 7 days [16], and it might begin to shed enterovirus prior to symptom onset, we speculated that the index case or someone else was the source of infection in this kindergarten [17].

Kindergartens are a highly susceptible location for HFMD outbreaks because large groups of children with close contact could create an excellent incubation site for HFMD. Children are easily infected and more likely easily to spread this disease [18]. In this outbreak, HFMD rapidly spread across 10 of the 11 classes within 11 days. It ceased after the HFMD control measures were implemented by SCDC. We speculated that at least two factors contributed to this rapid transmission. First, the interaction among children from different classes could accelerate the spread of the agent. Second, according to our previous study, some children can transmit the virus prior to symptom onset [17], and infected patients can excrete the virus for a long time. Given the highly infectious characteristics of HFMD, we strongly suggested that cases should be isolated and that thorough environmental disinfection be performed immediately once a case appeared.

As is known, EV-A71 vaccines are available in mainland China, and the protective efficacies are higher than 90% against EV-A71-associated HFMD [19]. However, these vaccines cannot protect people from suffering from HFMD caused by CVA6 [20]. In the absence of any effective vaccine or antiviral therapy, primary prevention is the most effective measure to prevent the outbreak of CVA6-associated HFMD.

A previous study has reported that different genetic background strains may cause different clinical manifestations of HFMD [21]. Confirmed by the VP1 gene sequencing, our results suggested that this outbreak was caused by the same CVA6. In this study, five out of ten CVA6-positive HFMD cases developed onychomadesis. Based on the results of this study, we proposed that the difference in clinical presentations might be correlated with the host background apart from the causative pathogen. Moreover, we found that patients with symptoms of desquamation had a 9.3-time chance of experiencing onychomadesis comparing with cases without desquamation. Combining the fact that desquamation in most cases appeared earlier than onychomadesis, we suggest that desquamation might be used as an indicator of developing onychomadesis. Based on the current situation that parents and even some physicians know little about HFMD related onychomadesis, misdiagnosis could happen. We hope that this study can provide evidence for the early treatment of HFMD and help address the concerns of the parents.

There are several limitations. First, we did not assess the effectiveness of individual control measure. Second, the data collection was telephone-based and recall bias might exist. Third, due to limited knowledge about the late symptoms of HFMD, the appearance of desquamation, onychomadesis or pigmentation might be underestimated.

## Conclusion

In summary, the 2015 Beijing HFMD outbreak was caused by CVA6, featured with delayed symptoms. Health care providers and parents need to pay more attention and raise awareness towards the CVA6-HFMD to be able to better control and prevent future disease outbreak.

## Additional file

Additional file 1: Onychomadesis distribution of finger nails affected per cases. (DOCX 21 kb)

## Abbreviations

CDC: Center for Disease Control and Prevention
CRP: C-reactive protein
CVA16: Coxsackievirus A16
CVA6: Coxsackievirus A6
EV-A71: Human enterovirus 71
HFMD: Hand, foot and mouth disease
WBC: White blood cell
