# Supplementary material for: An outbreak of Coxsackievirus A6–associated hand, foot, and mouth disease in a kindergarten in Beijing in 2015
Source: BMC Pediatr. 2018 Aug 21;18:277. doi: 10.1186/s12887-018-1253-1 (PMC6103857; doi:10.1186/s12887-018-1253-1)
Supplement: Supplementary file 1 — Onychomadesis distribution of finger nails affected per cases. (DOCX 21 kb) [file 12887_2018_1253_MOESM1_ESM.docx]

Additional file 1: Onychomadesis distribution of finger nails affected per cases.

| ID | Hand total | Lhn1 | Lhn2 | Lhn3 | Lhn4 | Lhn5 | Rhn1 | Rhn2 | Rhn3 | Rhn4 | Rhn5 | Feet total | Lfn1 | Lfn2 | Lfn3 | Lfn4 | Lfn5 | Rfn1 | Rfn2 | Rfn3 | Rfn4 | Rfn5 |
| --- | --- | --- | --- | --- | --- | --- | --- | --- | --- | --- | --- | --- | --- | --- | --- | --- | --- | --- | --- | --- | --- | --- |
| 1 | 2 | 1 | 0 | 0 | 0 | 0 | 1 | 0 | 0 | 0 | 0 | 2 | 1 | 0 | 0 | 0 | 0 | 1 | 0 | 0 | 0 | 0 |
| 4 | 1 | 0 | 0 | 0 | 0 | 0 | 0 | 0 | 1 | 0 | 0 | 0 | 0 | 0 | 0 | 0 | 0 | 0 | 0 | 0 | 0 | 0 |
| 7 | 4 | 1 | 1 | 0 | 0 | 0 | 1 | 1 | 0 | 0 | 0 | 0 | 0 | 0 | 0 | 0 | 0 | 0 | 0 | 0 | 0 | 0 |
| 8 | 1 | 0 | 0 | 0 | 1 | 0 | 0 | 0 | 0 | 0 | 0 | 0 | 0 | 0 | 0 | 0 | 0 | 0 | 0 | 0 | 0 | 0 |
| 11 | 1 | 1 | 0 | 0 | 0 | 0 | 0 | 0 | 0 | 0 | 0 | 1 | 1 | 0 | 0 | 0 | 0 | 0 | 0 | 0 | 0 | 0 |
| 12 | 3 | 1 | 0 | 0 | 0 | 0 | 1 | 0 | 1 | 0 | 0 | 0 | 0 | 0 | 0 | 0 | 0 | 0 | 0 | 0 | 0 | 0 |
| 13 | 2 | 1 | 0 | 0 | 0 | 0 | 1 | 0 | 0 | 0 | 0 | 0 | 0 | 0 | 0 | 0 | 0 | 0 | 0 | 0 | 0 | 0 |
| 15 | 2 | 1 | 0 | 1 | 0 | 0 | 0 | 0 | 0 | 0 | 0 | 0 | 0 | 0 | 0 | 0 | 0 | 0 | 0 | 0 | 0 | 0 |
| 16 | 3 | 1 | 1 | 0 | 0 | 0 | 1 | 0 | 0 | 0 | 0 | 0 | 0 | 0 | 0 | 0 | 0 | 0 | 0 | 0 | 0 | 0 |
| 18 | 2 | 0 | 1 | 0 | 0 | 0 | 0 | 1 | 0 | 0 | 0 | 2 | 1 | 0 | 0 | 0 | 0 | 1 | 0 | 0 | 0 | 0 |
| 23 | 3 | 1 | 0 | 1 | 0 | 0 | 0 | 1 | 0 | 0 | 0 | 0 | 0 | 0 | 0 | 0 | 0 | 0 | 0 | 0 | 0 | 0 |
| 25 | 10 | 1 | 1 | 1 | 1 | 1 | 1 | 1 | 1 | 1 | 1 | 0 | 0 | 0 | 0 | 0 | 0 | 0 | 0 | 0 | 0 | 0 |
| 27 | 2 | 1 | 1 | 0 | 0 | 0 | 0 | 0 | 0 | 0 | 0 | 0 | 0 | 0 | 0 | 0 | 0 | 0 | 0 | 0 | 0 | 0 |
| 32 | 2 | 1 | 0 | 0 | 0 | 0 | 1 | 0 | 0 | 0 | 0 | 1 | 0 | 0 | 0 | 0 | 0 | 1 | 0 | 0 | 0 | 0 |
| 33 | 2 | 1 | 0 | 0 | 0 | 0 | 0 | 1 | 0 | 0 | 0 | 0 | 0 | 0 | 0 | 0 | 0 | 0 | 0 | 0 | 0 | 0 |
| 34 | 5 | 1 | 1 | 1 | 1 | 1 | 0 | 0 | 0 | 0 | 0 | 0 | 0 | 0 | 0 | 0 | 0 | 0 | 0 | 0 | 0 | 0 |
| 38 | 2 | 1 | 0 | 0 | 0 | 0 | 1 | 0 | 0 | 0 | 0 | 0 | 0 | 0 | 0 | 0 | 0 | 0 | 0 | 0 | 0 | 0 |
| 41 | 10 | 1 | 1 | 1 | 1 | 1 | 1 | 1 | 1 | 1 | 1 | 0 | 0 | 0 | 0 | 0 | 0 | 0 | 0 | 0 | 0 | 0 |
| 43 | 4 | 1 | 1 | 0 | 0 | 0 | 1 | 1 | 0 | 0 | 0 | 6 | 1 | 1 | 1 | 0 | 0 | 1 | 1 | 1 | 0 | 0 |
| 44 | 2 | 1 | 0 | 0 | 0 | 0 | 1 | 0 | 0 | 0 | 0 | 2 | 1 | 0 | 0 | 0 | 0 | 1 | 0 | 0 | 0 | 0 |
| 45 | 6 | 1 | 1 | 0 | 1 | 0 | 1 | 1 | 0 | 1 | 0 | 2 | 1 | 0 | 0 | 0 | 0 | 1 | 0 | 0 | 0 | 0 |
| 52 | 0 | 0 | 0 | 0 | 0 | 0 | 0 | 0 | 0 | 0 | 0 | 1 | 1 | 0 | 0 | 0 | 0 | 0 | 0 | 0 | 0 | 0 |

Lhn1: the thumb of the left hand. Lhn2: the forefinger of the left hand. Lhn3: the middle finger of the left hand. Lhn4: the ring finger of the left hand. Lhn5: the little finger of the left hand. Rhn1: the thumb of the right hand. Rhn 2: the forefinger of the right hand. Rhn 3: the middle finger of the right hand. Rhn 4: the ring finger of the right hand. Rhn 5: the little finger of the right hand. Lfn1: the big toe of the left foot. Lfn2: the index toe of the left foot. Lfn3: the middle toe of the left foot.Lfn4: the fourth finger of the left foot. Lfn5: the little toe of the left foot. Rfn1: the big toe of the right foot. Lfn2: the index toe of the right foot. Lfn3: the middle toe of the right foot.Lfn4: the fourth finger of the right foot. Lfn5: the little toe of the right foot.
